# Renin dependent hypertension caused by accessory renal arteries

**DOI:** 10.1186/s40885-018-0100-x

**Published:** 2018-11-01

**Authors:** Pei Lin Chan, Florence Hui Sieng Tan

**Affiliations:** Department of Medicine, Sarawak General Hospital, Ministry of Health Malaysia, Jalan Hospital, 93586 Kuching, Sarawak Malaysia

**Keywords:** Accessory renal artery, Secondary hyperaldosteronism, Renovascular hypertension

## Abstract

**Background:**

Hypokalemia in the presence of hypertension is often attributed to primary hyperaldosteronism as a cause of secondary hypertension, however secondary hyperaldosteronism may present similarly. Accessory renal arteries are variants in the vascular anatomy which are often thought to be innocuous but in some circumstances can cause renovascular hypertension leading to secondary hyperaldosteronism.

**Case presentation:**

We report 2 cases of hypertension with secondary hyperaldosteronism associated with accessory renal arteries. Both patients presented with hypokalemia and further investigations revealed hyperaldosteronism with unsuppressed renin levels. Imaging studies showed the presence of accessory renal artery.

**Conclusion:**

Accessory renal arteries are a potential cause renovascular hypertension which can be detected via CT angiography or magnetic resonance angiography. Hormonal evaluation should be undertaken to determine whether its presence contributes to hypertension in the patient as targeted treatment such as aldosterone antagonist can be initiated. Surgical intervention or renal denervation may be considered in resistant cases.

## Background

Secondary hypertension occurs in 5–10% of the population and is important to identify as the cause is often treatable [1]. The investigation of secondary hypertension is guided by clinical clues. Patients with hypertension associated with hypokalemia are considered for further evaluation of primary hyperaldosteronism. However similar findings can occur in secondary hyperaldosteronism which is caused by reduced renal perfusion leading to the activation of the renin aldosterone system. Renal artery stenosis is the commonest cause of such pathology but a less well known cause is the presence of accessory renal arteries. Below we report 2 cases of hypertension with secondary hyperaldosteronism due to accessory renal arteries.

## Case presentation 1

**Fig. 1:**
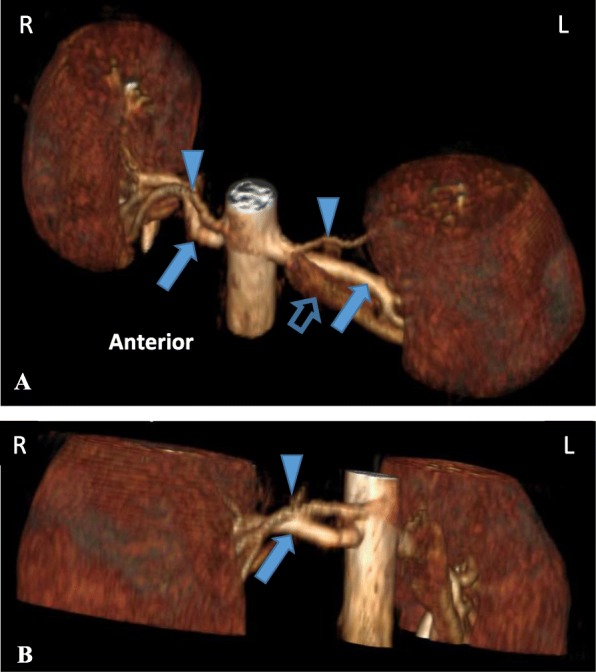
3-dimensional reconstruction of contrasted renal MRA. (**a**) Left anterosuperior oblique view showing bilateral accessory renal arteries arising above the main renal arteries (**b**) Right oblique view demonstrating the right renal accessory artery. (Arrowhead = accessory renal artery; closed arrow = main renal artery; open arrow = renal vein)

A 21 year old woman with no prior medical illness presented with epistaxis and raised blood pressure of 200/142 mmHg. She consumes 20 unit of alcohol per week and is a smoker of 1 pack year. On examination, she was obese with a body mass index (BMI) of 29.7 kg/m^2^. Physical examination was otherwise unremarkable with no hirsutism nor cushingnoid features. There was no abdominal bruit, radio-radial, or radio-femoral delay. Laboratory investigation at presentation showed hypokalemia (potassium 2.6 mmol/L) and alkalosis. Renal function, liver function, thyroid function, fasting blood glucose and lipid profile were within normal limits. 8 am cortisol was 17.11 μg/dL. Echocardiography showed asymmetrical left ventricular hypertrophy. She was treated with prazosin 2 mg tds and amlodipine 10 mg daily as well as oral potassium chloride 1.2 g od to maintain normal blood pressure and potassium level. Further work up after normalization of potassium revealed secondary hyperaldosteronism with elevated plasma aldosterone 1100 pmol/L (Reference range 102–858) and direct plasma renin 230.10 mIU/L (Reference range 4.2–59.7); giving a aldosteorone renin ratio (ARR) of 5 pmol/mIU. There was no evidence of renal artery stenosis on renal Doppler study. Renal magnetic resonance angiography (MRA) showed normal renal arteries bilaterally but bilateral accessory renal arteries were seen superior to the main renal arteries (Fig. 1). Renal angiography had no evidence of stenosis in the main or the accessory arteries bilaterally. In view of the absence of demonstrable stenosis for intervention, the patient was put on medical therapy. Her blood pressure was subsequently controlled on spironolactone 75 mg daily and amlodipine 10 mg daily.

## Case presentation 2

**Fig. 2:**
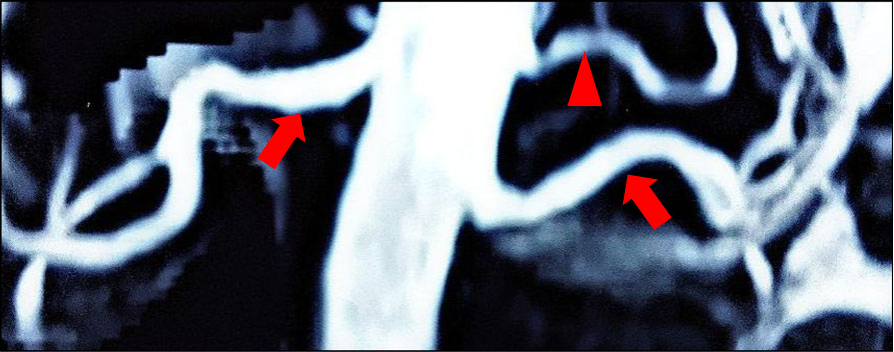
Renal MRA. MR angiography of the renal arteries in a 40-year-old woman with hypertension and secondary hyperaldosteronism demonstrating a nonstenotic left accessory renal artery. (Arrowhead = accessory renal artery; closed arrow = main renal artery)

A 41 year old lady with history of hypertension for 3 years treated with amlodipine 5 mg daily, presented with body weakness for a week and difficulty climbing stairs for a few months. She did not have any prior gastrointestinal losses and denied the use of traditional medications. Investigation done showed hypokalemia at 1.8 mmol/L and she was hospitalized. On examination, blood pressure was 145/100 mmHg with pulse rate of 85 per minutes. Her BMI was 30.5 kg/m^2^ but she did not appear cushingnoid. There was no abdominal bruit. Physical examination was otherwise unremarkable. Investigation showed normal thyroid function, renal function and normal serum calcium and magnesium. There was metabolic alkalosis with serum bicarbonate of 32 mmol/L. Cortisol after overnight 1 mg dexamethasone suppression was normal at 0.69 μg/dl. Hypertension was controlled with diltazem 30 mg tds but she required oral potassium chloride at 1.8 g tds to maintain normal potassium level. Serum aldosterone was 1046 pmol/L with plasma renin of 6.5 ng/ml/hour (reference range 0.2–2.8) giving an ARR of 161. Her echocardiogram was normal with no left ventricular hypertrophy or coarctation of aorta. Renal Doppler showed prolonged acceleration time of the left renal artery with spectral widening. Peak systolic velocities and resistive indices within normal limits but findings were suspicious for left renal artery stenosis. MRA of the kidneys revealed normal kidneys and normal main renal artery calibers bilaterally. However a small accessory left renal artery was seen 1 cm above the origin of the left main renal artery supplying the upper pole (Fig. 2). There was no stenosis detected in the accessory artery. Her blood pressure and hypokalemia were controlled with spironolactone 50 mg daily and oral potassium chloride 1.2 g daily.

## Discussion

We described two females with young hypertension and spontaneous hypokalemia in whom work-up for primary hyperaldosteronism revealed elevated renin and aldosterone level consistent with secondary hyperaldosteronism. Imaging studies with MRA however did not show stenosis or fibromuscular dysplasia of the renal artery but instead demonstrated the presence of accessory renal arteries.

Accessory renal arteries are common and may be present in 20–60% of population [2–4]. These are extra arteries that supply the renal hilum and are also known as supernumerary renal arteries. Its association to renovascular hypertension had been described as early as 1951 [3] with several studies showing increase presence of multiple renal arteries in hypertensive versus normotensive patients [5]. Györi hypothesized that accessory renal artery can lead to hypertension due to under perfusion of the kidney as a result of longer and narrow caliber which raises its resistance or predisposes it to stenosis [6]. The resultant renal ischemia lead to activation of renin-angiotensin system and secondary hypertension. Glodny demonstrated increased renin activities in patients with multiple renal arteries compared to those with single renal artery [7]. On the contrary, there are studies which found no association of accessory renal arteries to hypertension [8, 9]. This is not surprising given the marked variation in anatomy, size and hemodynamic contribution of each accessory artery to the renal vasculature as well as differences in the method of assessment and population studied. Convincing evidence of accessory renal arteries causing renin dependant renovascular hypertension have rarely been reported in the literature. From our literature search, only 3 such cases with hormonal evaluation were found. These cases are summarized in Table 1.

**Table 1:** Summary of case reports previously reported in the literature

	Patient 1 (reference [15])	Patient 2 (reference [15])	Patient 3 (reference [21])
Clinical	Patient with severe hypertension (BP 190/130 mmHg) partially controlled with 2 antihypertensive medications.	Young adolescent with uncontrolled hypertension (BP 220/115 mmHg) treated with beta blocker and diuretic.	Uncontrolled hypertension in a young patient investigated for secondary hypertension.
Hormonal studies	PA 15 ng/dL PRA 8 ng/ml/hour ARR 1.8	PA 23 ng/dL PRA 18 ng/ml/hour ARR 1.3	Supine renin 400 pg/mL (Normal 2.4–21.9)
Selective renal vein sampling	Renin vein (right/left) ratio 4.3:1 after captopril	Renin vein (right/left) ratio 8:1 after captopril	
Imaging	Arteriogram showed elongated, nonstenotic aberrant artery arising from the common iliac artery supplying the lower pole of the right kidney	Arteriogram showed nonstenotic aberrant artery arising from the lower aorta feeding the lower pole of the left kidney	Digital subtraction angiography showed small (2-mm) left accessory RA entrapped by the diaphragmatic crus with 90% proximal ostial segment stenosis
Medication	Propranolol 50 mg twice per day and hydrochlorothiazide 50 mg/d	Metoprolol 50 mg twice per day and hydrochlorothiazide 50 mg a day	Atenolol 50 mg & amlodipine 10 mg daily
Outcome	BP 120/70 mmHg off antihypertensive after left partial nephrectomy.	Medical therapy with captopril, diuretic and beta blocker. Subsequently lost to follow up.	Decision was made for medical therapy

*PA* plasma aldosterone expressed in ng/dl [conversion factor to SI (pmol/L = 27.741], *PRA* plasma renin activity

Various imaging methods have been used to further assess patients with renovascular hypertension. The detection of accessory renal artery is better with the use of CT angiography or gadolinium enhanced MRA compared to Doppler ultrasound [10].To further evaluate the hemodynamic significance of a stenotic vessel, renal vein renin sampling as well as radionuclide imaging such as captopril renogram have been used though its role in accessory renal artery have only been reported in a few case reports [11–13]. Treatment included medical therapy, or stenting in the presence of stenosis or fibromuscular dysplasia [14] and nephrectomy [15] in patient who failed medical therapy.

More recently, the finding of accessory renal arteries was highlighted in cases of treatment resistant hypertension undergoing sympathetic renal denervation. Studies have found higher rates of nonresponders or less pronounced blood pressure reduction in those with accessory renal arteries compared to those with bilateral single renal arteries, especially if the accessory arteries were not treated [16–19], again supporting the possibility of their contributing role in hypertension. Newer technologies that are able to target accessory arteries for more complete renal denervation may offer another treatment option for patients with resistant hypertension [19, 20].

## Conclusion

In conclusion, accessory renal arteries are common and depending on the anatomy and hemodynamic characteristics may contribute to resistant hypertension through renin dependent hyperaldosteronism. Treatment include medical therapy with aldosterone antagonist but in resistant cases surgical intervention or perhaps renal denervation may be considered.

## Abbreviations

ARR: Aldosterone renin ratio
BMI: Body mass index
MRA: Magnetic resonance angiography
PA: Plasma aldosterone
PRA: Plasma renin activity
